# Supplementary material for: Brain and circulating steroids in an electric fish: Relevance for non-breeding aggression
Source: PLoS One. 2023 Oct 10;18(10):e0289461. doi: 10.1371/journal.pone.0289461 (PMC10564164; doi:10.1371/journal.pone.0289461)
Supplement: Attachment — Submitted filename: Zubizarreta Jalabert et al response to reviewers.docx [file pone.0289461.s001.docx]

Irfan Ahmad Bhat

Academic Editor

PLOS ONE

Dear Dr. Irfan Ahmad Bhat

Please find attached the revised manuscript “Brain and circulating steroids in an electric fish: relevance for non-breeding aggression” authored by Lucia Zubizarreta, Cecilia Jalabert (equal contributors) Ana Silva, Kiran K. Soma and myself both as corresponding authors, to be considered for its publication as an original research article.

We thank the reviewers for their comments and suggestions that allowed us to greatly improve this manuscript. We have addressed all comments point by point, as detailed below (changes in the revised manuscript are in blue).

In summary, we made 3 major changes. First, we included more information on the mechanisms underlying the regulation of non-breeding aggression, discussing the role of progestogens and estrogens, as well as non-steroidal molecules. Second, we clarified the methods section, including details of the internal standards and the statistics employed. Third, we have improved the quality of the figures, which now meet the standards of the journal.

In addition, we have taken into account the evaluation of reviewer 1 and revised the manuscript throughout to comply with standard English.

As indicated in the decision letter, we would be grateful if you could make the following amendments:

a. Since we have no ethical or legal restrictions on sharing our data set, we would like our Data Availability statement to be amended. We have uploaded the database needed to replicate the findings of this study in a public repository (<https://doi.org/10.17605/OSF.IO/W8ZMJ>).
b. In the Role of Funder statement please add: "The funders had no role in study design, data collection and analysis, decision to publish, or preparation of the manuscript."
c. In the Competing Interests statement please add: "The authors have declared that no competing interests exist."

Sincerely yours,

Laura Quintana

Assistant Professor

Unidad Bases Neurales de la Conducta, Departamento de Neurofisiología Celular y Molecular

Reviewer #1: Authors have done a great job of investigating the plasma and brain steroids in electric fish and how they influence nonbreeding aggression. I have a few comments. Please find them below.

1. Why were the individuals collected only during non-breeding season? Authors should consider breeding aggression based on sexual differences in fishes.

**Response: Our aim was specifically to study the steroid profiles present in the non-breeding season in a species that shows robust territorial aggression during this season. Non-breeding aggression is a useful behavior to understand neuroendocrine mechanisms that differ from those underlying breeding aggression (which is most commonly studied). In the non-breeding season, circulating sex steroids are often at low concentrations, offering a “clean field” to study sex steroids in the brain. We have added a mention of this in the abstract (lines 38-41). This paradigm has been key in revealing the brain as a source of steroids that regulate aggression not only in fish, but also in birds and mammals. In addition, these models strongly suggest a common role of neuroestrogens in the control of aggression during this season. Interestingly, in *Gymnotus omarorum*, this sexually monomorphic behavior may stem from sexually dimorphic hormonal actions, as suggested by our results. This is an interesting avenue to research, as it may shed light on how sexually-biased mechanisms may lead to the same outcomes. The study of breeding hormonal profiles and behavior is not part of this study, although it is currently an area of research in our lab and will be the focus of future publications.**

2. Authors should consider this manuscript while writing particular statements <https://www.jneurosci.org/content/41/44/9177>

**Response: Thank you for this comment. Wartenberg et al. beautifully show neurosteroidal influences during development that are ultimately related to sexual dimorphism in aromatase-expressing neurons at birth. The spatiotemporal data, obtained in ArIC/eR26-τGFP reporter mice, compares embryos to neonates to shed light on novel mechanisms of estrogenic regulation during development. In our manuscript, we state that the sex differences in hormonal profiles may be due to “...ii) sex differences in brain steroidogenic enzymes that synthesize or metabolize steroids…” (line 406), which encompasses the data in the aforementioned paper, and we have now added it to our cited references.**

3. Authors have quantified only androgens and estrogen, why not progesterone metabolites using LCMS/MS.

**Response: Progesterone was included in our panel of steroids to be quantified by LC-MS/MS (mentioned in our original manuscript, now in lines 198 and 199). However, progesterone was not detectable in either plasma or brain samples in both sexes (stated in lines 290 and 307). We agree with the referee that the role of progestogens in aggression is very important, and it is now discussed in lines 383-389. This is the first time circulating and brain steroids have been measured in this species, and we did not include the quantification of progesterone metabolites in this first study, as acknowledged in the original manuscript (and the current one in lines 387-389).**

4. Authors have used different internal standard “deuterated internal standards (progesterone-d9, cortisol-d4, DHEA-d6, testosterone-d5, E2- 146 d4; C/D/N Isotopes Inc., Pointe-Claire, Canada)”. Can they briefly mention which one was used for which compound and their retention time details?

**Response: Thank you for this observation. In our original manuscript, we stated retention time details for all analytes and internal standards (specified in Table 1). We have now clarified which internal standard was used for each analyte in the methods section (lines 148-150).**

5. Can you please share in detail the method development for the brain steroids?

**We are not sure what the reviewer refers to in this point. We understand that the description of the method in the manuscript was already detailed enough to be replicated: extraction method (lines 141-170), LC-MS/MS method (lines 173-190), and validations (lines 208-218). The changes made addressing the previous point have added important details to our method description.**

6. Authors should combine discussion into a single heading, instead of making different sub heading.

**Response: Thank you. We have carefully considered this suggestion, and we find that the three sub-headings help organize our different lines of discussion.**

7. Please improve the resolution of figures.

**Response: We are grateful for this observation and have corrected the figures accordingly.**

Reviewer #2: The manuscript is well written. However, I have some minor queries which should be addressed.

Authors should mention how neurosteroids are modulating fish behavior?

**Response: We have now included this point in the final part of the discussion.**

Is aggression mainly attributed due to steroids only or there are some other factors as well that needs to be mentioned in the manuscript?

**Response: Thank you for this comment. The neuropeptide arginine vasotocin (AVT) has a rapid status-dependent effect on non-breeding aggressive behavior, and serotonin also modulates aggression. We have added this information to the introduction (lines 94-96).**

Statistical analysis should be rewritten, it bit unclear.

**Response: We have revised the Statistics section to make it clearer.**

Authors should increase the visibility of figures?

**Response: We are grateful for this observation and have corrected the figures accordingly.**
